# “Living Well with Chronic Pain”: Integrative Pain Management via Shared Medical Appointments

**DOI:** 10.1093/pm/pnaa418

**Published:** 2021-02-04

**Authors:** Josie Znidarsic, Kellie N Kirksey, Stephen M Dombrowski, Anne Tang, Rocio Lopez, Heather Blonsky, Irina Todorov, Dana Schneeberger, Jonathan Doyle, Linda Libertini, Starkey Jamie, Tracy Segall, Andrew Bang, Kathy Barringer, Bar Judi, Jane Pernotto Ehrman, Michael F Roizen, Mladen Golubić

**Keywords:** Chronic Pain, Opioids, Pain Management

## Abstract

**Objective:**

To evaluate the effectiveness of a multidisciplinary, nonpharmacological, integrative approach that uses shared medical appointments to improve health-related quality of life and reduce opioid medication use in patients with chronic pain.

**Design:**

This is a retrospective, pre–post review of “Living Well with Chronic Pain” shared medical appointments (August 2016 through May 2018).

**Setting:**

The appointments included eight 3-hour-long visits held once per week at an outpatient wellness facility.

**Subjects:**

Patients with chronic, non–cancer-related pain.

**Methods:**

Patients received evaluation and evidence-based therapies from a team of integrative and lifestyle medicine professionals, as well as education about nonpharmacological therapeutic approaches, the etiology of pain, and the relationship of pain to lifestyle factors. Experiential elements focused on the relaxation techniques of meditation, yoga, breathing, and hypnotherapy, while patients also received acupuncture, acupressure, massage, cognitive behavioral therapy, and chiropractic education. Patients self-reported data via the Patient-Reported Outcomes Measurement Information System (PROMIS-57) standardized questionnaire. Use of opioid medications was evaluated in morphine milligram equivalents.

**Results:**

A total of 178 participants completed the PROMIS-57 questionnaire at the first and the last visits. Statistically significant improvements in all domains (Physical Functioning, Anxiety, Depression, Fatigue, Social Roles, Pain Interference, and Sleep Disturbance) were observed (*P* < 0.001) between the pre-intervention (visit 1) and post-intervention (visit 8) scores. Average opioid use decreased nonsignificantly over the 8-week intervention, but the lower rate of opioid use was not sustained at 6 and 12 months’ follow-up.

**Conclusions:**

Patients suffering from chronic pain who participated in a multidisciplinary, nonpharmacological treatment approach delivered via shared medical appointments experienced reduced pain and improved measures of physical, mental, and social health without increased use of opioid pain medications.

## Introduction

Chronic pain affects more than 100 million Americans each year, more than are affected by heart disease, stroke, cancer, and diabetes combined. An estimated $635 billion each year is spent on direct medical costs, lost productivity, and disability programs for patients suffering from chronic pain. Almost 20 million adults suffer from high-impact pain severe enough to interfere with life or work activities on most days [1]. During the past few decades, an overly enthusiastic focus on using medications for pain management contributed to an epidemic of opioid-related deaths that has recently reached unparalleled proportions [2]. The Centers for Disease Control and Prevention reported a total of 63,632 opioid overdose deaths in 2017 in the United States from both prescription and illegal opioids, with nearly half of these deaths attributed to prescription opioid treatment for pain [3, 4].

In 2018, the Joint Commission provided guidance for evidence-based, non-opioid treatment options that can be considered for treating pain, including behavioral therapies, meditation techniques, acupuncture, spinal manipulation, massage, and music therapy [5]. Recent guidelines from the American College of Physicians [6, 7] similarly recommend exercise, multidisciplinary rehabilitation, mindfulness-based stress reduction, acupuncture, tai chi, yoga, and spinal manipulation as nonpharmacological treatment options for patients with chronic low back pain. Although the evidence for use of these nonpharmacological therapies is compelling and increasing [8–13], these pain relief approaches are rarely reimbursed by medical insurance. Furthermore, many medical facilities and practices lack properly trained and certified practitioners to deliver these nonpharmacological, lifestyle, integrative, and complementary therapeutic modalities. A focus on implementation of integrative multimodality approaches is essential in light of guidelines that call for a systemized approach to review *all* aspects of a patient’s situation while focusing on symptomatic relief.

Shared medical appointments (SMAs) are increasingly used to improve the health and daily living of patients with common chronic diseases, such as cardiovascular disease, obesity, diabetes, and breast cancer [14–18]. It has been reported previously that patients with chronic pain participating in the SMAs were treated with integrative medical modalities, including mindfulness practices, self-massage, acupuncture, and similar approaches, as education only [19], education plus an individual integrative modality [20], or a combination of different therapies [21–25]. Improvements in pain severity, depression, and quality of life, as well as reduced pain medication use and fewer emergency department visits, were documented. In the present study, we report outcomes for patients with chronic pain who participated in the “Living Well with Chronic Pain” SMAs and were treated in a comprehensive fashion with a broad spectrum of evidence-based integrative and self-care therapies.

## Methods

The present investigation was conducted from August 2016 through May 2018 at an outpatient facility of the Cleveland Clinic, at the Centers for Integrative and Lifestyle Medicine. It was reviewed and approved by the Institutional Review Board (IRB#16–671).

### Study Design

The Cleveland Clinic “Living Well with Chronic Pain” SMAs occurred over 8 weeks (with a single 3-hour visit per week) and were facilitated by a physician and a holistic psychotherapist. Providers of a particular integrative modality participated in and facilitated individual group appointments when their therapeutic approach was featured. The SMA format (8–13 participants) allows for social reintegration and community building and is a safe environment in which to practice new skills. The goals of these SMAs were to help individuals learn and develop techniques to aid in decreasing the sensations of pain in the physical body and to help manage the emotional effects of living with chronic pain. We used tools to assist participants with pain and stress relief, lifestyle modifications, and positive behavioral changes. The group visits enabled participants to determine the tools that worked best for them, eliminate or limit negative thoughts and behaviors, and create their own specialized plan of care on the basis of the 8 weeks of practice and exposure to new ways of managing pain.

In this study, each 3-hour weekly session included measurement of patient vitals and physical assessment, individual check-in with the physician and the group, a topic lecture, self-massage, gentle chair yoga, auricular acupuncture, and hypnotherapy/meditation. Topic lectures were focused on acupuncture and acupressure for pain reduction, massage, nutrition and supplementation, chiropractic education about proper techniques for performing activities of daily living, mechanisms of action of pain, art therapy or guided imagery for emotional wellness, goal setting, and Chinese herbal medicine. Patients were encouraged to pursue individual follow-up appointments in an integrative or lifestyle medicine area they found beneficial. Additionally, monthly group follow-up sessions were held to support lifestyle changes and encourage healthy habits.

### Patient Enrollment

**Figure 1.**
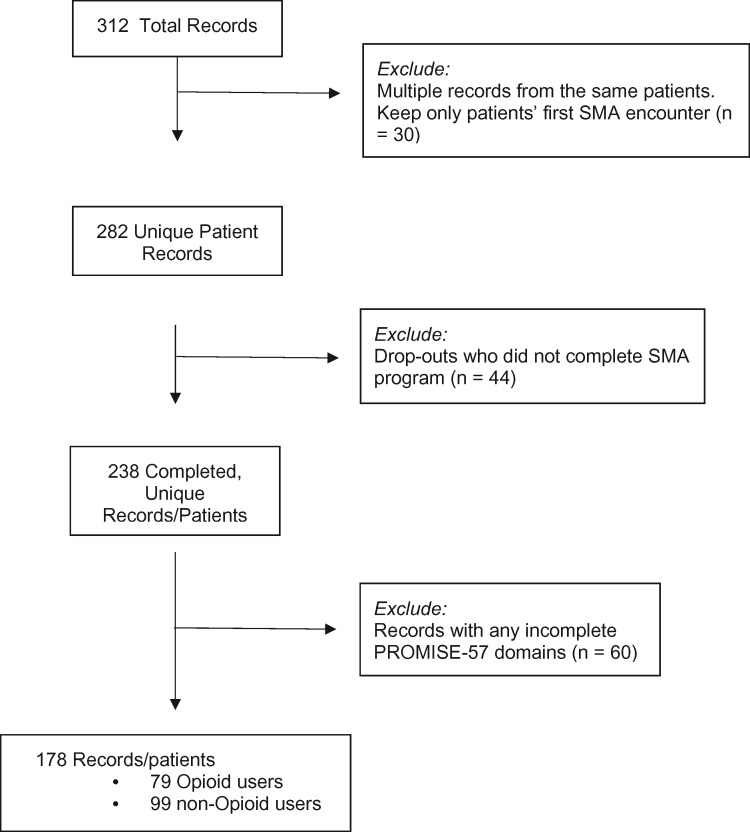
CONSORT diagram of participant flow.

Patients included in this study were referred by their primary care physicians as anyone self-reporting as living with chronic pain, independent of the specific diagnosis or prior treatment involvement. This study accepted patients who may have undergone different forms of treatment, including opioid prescription medication. The SMA providers were informed of but did not manage, initiate, or discontinue pain medications, nor were they involved in the diagnostic work-up for the source of pain sensations. Patients were included in this study if they completed both pre-evaluation (the first SMA, week 1) and post-evaluation (the last SMA, week 8) Patient Reported Outcome Measurement Information System (PROMIS-57) surveys. Patients starting the SMAs after August 1, 2018, were not considered because they would not have had a full year of follow-up with regard to their opioid use evaluation. As such, a total of 178 patients (out of 312 patients enrolled) met these criteria (Figure 1). Among those, there were 79 opioid users and 99 non-opioid medication users. For patients (n = 30) who participated in multiple sessions of the 8-week SMAs during the study period, only pre/post data from their first set of SMAs were included in analysis.

### Pain SMA Program Activities

**Figure 2.**
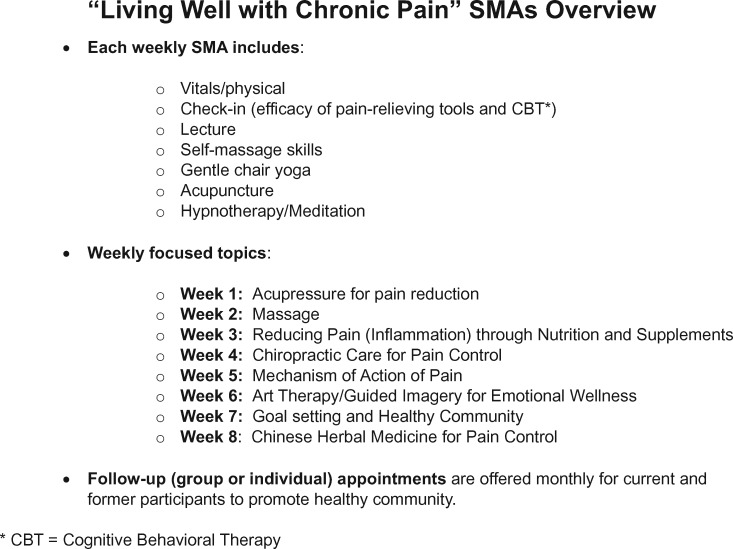
Overview of the group visit structure of the “Living Well with Chronic Pain” SMAs.

An overview of the group visit structure of the “Living Well with Chronic Pain” SMAs is depicted in Figure 2.

#### Patient Vitals and Physical Assessment

SMA providers performed a brief physical assessment of each patient, which included measurement of heart rate and blood pressure, a brief examination, and an evaluation of general mental and physical well-being.

#### Check-in

The holistic psychotherapist–led check-in was an essential component of the program. Through a series of weekly questions, the aim of check-in was to see which tools each person was using and finding helpful. Check-in was a time for the holistic psychotherapist to listen deeply to the language of the participants and assist with cognitive restructuring and reframing of negative thought patterns (cognitive behavioral therapy techniques). The focus of check-in was to shift thoughts to a more positive outlook and to learn that the mind and body are connected.

#### Topic Lecture

Each week, a specific topic was presented as part of the SMA visit. Topics were designed and specifically chosen to reflect the tools to which patients would be exposed during the 8 weeks, including Korean Sujok-style acupressure for pain reduction, massage, nutrition and supplementation, chiropractic education, education about the mechanisms of action of pain, art therapy or guided imagery for emotional wellness, goal setting, and Chinese herbal medicine.

#### Massage

Participants were taught trigger-point release, gentle stretching, relaxation, effleurage, hot and cold applications, and cross-fiber friction.

#### Yoga

The program offered gentle chair yoga, which allows the participants to move their bodies in a simple way, learning to stretch and to become more aware of their physical sensations in the moment. The yoga was adapted for people of all abilities, and no prior yoga experience was necessary. Breathing and mindfulness techniques were an important part of this yoga practice.

#### Acupuncture

All participants received prescribed auricular acupuncture in a group setting at the end of each weekly session. While participants were treated with acupuncture, a holistic psychotherapist guided them in a hypnotherapy process to induce a state of relaxation in the body and decreased discomfort.

#### Hypnotherapy/Meditation

Affirmations and journaling prompts were provided to encourage positive self-talk, self-reflection, and healthy emotional expression.

The goal was to offer participants a wide variety of tools from which to choose. At the end of 8 weeks, participants were able to share their overall experience, both good and bad, but importantly of how the diverse skills and techniques learned throughout all SMA sessions had contributed to a higher quality of life and functionality. Participants were also encouraged to engage in individual holistic psychotherapy sessions throughout the program to gain additional support for what they had learned in the group visits, as well as to address social/emotional challenges.

### Data Collection

Primary study end points included the PROMIS-57 Profile v2.0, which contains a separate eight-question scale for each of seven domains: 1) Physical Function, 2) Anxiety, 3) Depression, 4) Fatigue, 5) Sleep Disturbance, 6) Ability to Participate in Social Roles and Activities, and 7) Pain Interference. Mean population scoring of each PROMIS domain is defined as having a value of 50 (standard deviation [SD] of 10; range, 0–100), with larger scores indicating greater amounts of each health element (100 = maximal physical function, anxiety, depression, etc.). In question 57 of the questionnaire, patients were also asked to rate their pain intensity on a 0 to 10 Likert pain scale that denotes 0 as representing “no pain” and 10 representing “the worst pain possible.”

Patient demographic data were collected at baseline, and all medications, including opioid prescription use in morphine milligram equivalents (MME), were recorded weekly throughout the 8-week intervention period. Data were collected either manually via paper forms or digitally via iPad and were entered into a protected, digital database (REDCap^®^ 9.1.20, Vanderbilt University).

### Statistical Analysis

Patient groups were described with medians and quartiles or mean ± SD for continuous variables and counts and percentages for categorical variables. PROMIS-57 raw scores were standardized to a mean of 50 and an SD of 10. Normality of data was assessed by evaluating QQ plots and model residuals for analysis of variance (ANOVA) and analysis of covariance (ANCOVA) analyses. Statistical analysis of the change in opioid MME dosage and PROMIS-57 standardized scores before and after SMAs was performed with paired *t* tests. Comparison between non-opioid and opioid users was done with ANOVA and ANCOVA; for the ANCOVA, the change in score was modeled as the outcome, with opioid use and the pre-SMA score as the independent variables. Results estimate the change from baseline and not the efficacy of the integrative medicine–based comprehensive nonpharmacological intervention. As a sensitivity analysis, analyses assessing changes in PROMIS-57 subscores and those comparing said changes between opioid and non-opioid users were repeated, including subjects who had incomplete PROMIS domains, as well as those who did not complete the SMA program. Missing baseline/final PROMIS-57 data were handled in two ways: 1) multiple imputation (five sets) for all missing values and 2) multiple imputation for pre-SMA missing values and last observation carried forward for post-SMA missing values; the latter (#2 above) assumes no change for all subjects who did not complete the post-SMA questionnaire and reflects the minimal average change. All tests were two-tailed and performed at a significance level of 0.05 in SAS 9.4 software (SAS Institute, Cary, NC).

## Results

### Characteristics of Study Participants

The description of the study cohort (n = 178) is shown in Table 1. The median age for all participants with chronic pain was 62 years. The majority of participants were female (87%), were white (69%), had post–high school education (90%), and were nonsmokers (58%) or past smokers (40%); 47% were retired. The median baseline body mass index for all participants was 30.9 (class I obesity). Opioid medication users (n = 79) comprised 44% of the total number of study participants. The attendance of patients who completed questionnaires at the first and the last group appointments of their weekly SMAs was high. About 80% of patients participated in 7 or 8 group appointments, with the remaining patients being present at 1 to 6 SMAs (Table 1). There were no significant differences in baseline characteristics between patients using and not using opioid medications.

**Table 1.** Baseline demographics for participants

Factor	Total (N = 178)	No Opioids (N = 99)	Opioids (N = 79)
Race,* n (%)			
White	120 (69)	68 (70)	52 (68)
Black or African American	44 (25)	25 (26)	19 (25)
American Indian or Alaska Native	2 (1)	2 (2)	0 (0)
Chinese	1 (0.6)	0 (0)	1 (1)
Other Asian	1 (0.6)	1 (1)	0 (0)
I prefer not to answer	6 (3)	1 (1)	5 (6)
Age,* median [Q1, Q3]	62.0 [53.0, 69.0]	60.0 [51.0, 69.0]	63.0 [55.0, 70.0]
Highest level of education completed,* n (%)			
Some high school	4 (2)	3 (3)	1 (1)
High school graduate or GED	14 (8)	8 (8)	6 (8)
Some college, no degree	39 (23)	17 (18)	22 (29)
Associate or 2-year technical degree / vocational school	25 (15)	13 (14)	12 (16)
Bachelor’s degree	53 (31)	29 (30)	24 (32)
Master’s degree	31 (18)	23 (24)	8 (11)
Doctorate degree	2 (1)	1 (1)	1 (1)
Professional degree	4 (2)	2 (2)	2 (3)
Current employment status,* n (%)			
Employed	47 (27)	30 (31)	17 (22)
Unemployed	45 (26)	27 (28)	18 (24)
Retired	80 (47)	39 (41)	41 (54)
Gender,* n (%)			
Male	23 (13)	12 (12)	11 (15)
Female	150 (87)	86 (88)	64 (85)
Total weeks attended, n (%)			
2	1 (0.6)	1 (1)	0 (0)
4	1 (0.6)	0 (0)	1 (1)
5	6 (3)	3 (3)	3 (4)
6	27 (15)	14 (14)	13 (16)
7	46 (26)	24 (24)	22 (28)
8	97 (54)	57 (58)	40 (51)
Tobacco use,* n (%)			
Past smoker	68 (40)	37 (38)	31 (41)
Nonsmoker	99 (58)	55 (57)	44 (59)
Current smoker	5 (3)	5 (5)	0 (0)
BMI,* median [Q1, Q3]	30.9 [26.1, 37.2]	30.1 [26.2, 37.5]	31.3 [25.9, 36.2]

* Data not available for all subjects. Missing values: race = 4, age = 4, highest level of education completed = 6, current employment status = 6, gender = 5, tobacco use: = 6, and body mass index (BMI) = 37.

### Changes in Pre–Post PROMIS-57 Scores

The baseline measurements in seven domains of the PROMIS-57 questionnaire and Pain Intensity for all study participants (N = 178) are shown in Table 2. All raw scores for individual domains except pain intensity were standardized to a mean of 50 and an SD of 10.

**Table 2.** PROMIS-57 questionnaire measurements at baseline and at the end of the SMAs (after)

PROMIS-57 Domain Subscore, Mean ± SD	Total (N = 178)	No Opioids (N = 99)	Opioids (N = 79)
Physical Function *t* score			
Baseline	36.6 ± 5.9	37.8 ± 6.1	35.1 ± 5.4
After	37.9 ± 6.5	39.2 ± 6.7	36.3 ± 5.8
Anxiety *t* score			
Baseline	58.0 ± 9.7	58.0 ± 9.2	57.8 ± 10.4
After	55.5 ± 8.7	55.2 ± 8.9	55.8 ± 8.4
Depression *t* score			
Baseline	53.8 ± 8.4	53.4 ± 8.6	54.4 ± 8.1
After	51.7 ± 8.5	51.3 ± 8.8	52.2 ± 8.1
Fatigue *t* score			
Baseline	60.9 ± 9.2	60.1 ± 9.1	61.9 ± 9.2
After	57.8 ± 9.5	56.0 ± 9.7	60.0 ± 8.7
Sleep Disturbance *t* score			
Baseline	55.6 ± 8.4	55.0 ± 8.7	56.5 ± 7.9
After*	53.5 ± 9.3	52.4 ± 9.8	54.8 ± 8.6
Social *t* score^†^			
Baseline	41.0 ± 6.8	42.1 ± 6.7	39.7 ± 6.8
After*	43.9 ± 7.7	45.6 ± 7.7	41.8 ± 7.2
Pain Interference *t* score			
Baseline	65.2 ± 5.9	64.5 ± 6.0	66.0 ± 5.6
After*	61.7 ± 7.4	60.7 ± 7.9	63.0 ± 6.6
Pain Intensity			
Baseline	6.2 ± 1.7	6.2 ± 1.8	6.3 ± 1.6
After*	5.2 ± 2.3	5.2 ± 2.3	5.2 ± 2.2

* Data not available for all subjects. Patients with incomplete data for only one domain on eight-item PROMIS-57: Sleep Disturbance *t* score (after) = 1, Social *t* score (after) = 2, Pain Interference *t* score (after) = 2, Pain Intensity subscore (after)=2.

† Pertains to “Ability to Participate in Social Roles and Activities” domain.

At the start of the SMAs, there were no significant differences between patients taking or not taking opioids in terms of PROMIS-57 domains. The pre–post changes in PROMIS-57 domain scores for all patients (N = 178) are shown in Table 3. Statistically significant improvements were observed at the end of the SMAs when compared with the first SMA in all PROMIS-57 domains (Physical Functioning, Anxiety, Depression, Fatigue, Ability to Participate in Social Roles and Activities, Pain Interference, and Sleep Disturbance) (*P* < 0.001) and in Pain Intensity (*P* < 0.001). In two PROMIS domains, Fatigue and Pain Interference, the changes in *t* score were above 3 (Table 3), while in four additional PROMIS-57 domains (Anxiety, Depression, Sleep Disturbance, and Ability to Participate in Social Roles and Activities), *t*-score improvements were higher than 2. As shown in Table 3, non-opioid users showed greater improvements in six of eight domains, but those changes were not significantly different from the group of participants who used opioid medications. After adjustment for pre-SMA scores, differences in the domains of Fatigue and Ability to Participate in Social Roles and Activities were statistically significant between groups. Sensitivity analyses incorporating excluded subjects showed consistent results across all scenarios (Supplementary Data).

**Table 3.** Comparison of changes in PROMIS-57 scores before and after SMAs among all patients, those without use of opioid medications, and those with use of opioid medications

PROMIS-57 Domain Subscore (Post – Pre), Mean (95% CI)*		Unadjusted			Adjusted		
	All	No Opioids	Opioids	*P* Value^§^	No Opioids	Opioids	*P* Value^¶^
	(N = 178)	(N = 99)	(N = 79)		(N = 99)	(N = 79)	
Physical Function	1.3 (0.79 to 1.9)^‡^	1.5 (0.74 to 2.2)	1.2 (0.35 to 2.0)	0.6	1.6 (0.86 to 2.3)	1.02 (0.19 to 1.9)	0.31
Anxiety	−2.5 (-3.5 to -1.4)^‡^	−2.8 (-4.2 to -1.4)	−2.0 (-3.6 to -0.40)	0.45	−2.8 (-4.0 to -1.6)	−2.0 (-3.4 to -0.66)	0.42
Depression	−2.1 (-3.01 to -1.3)^‡^	−2.1 (-3.3 to -0.91)	−2.2 (-3.5 to -0.88)	0.9	−2.2 (-3.3 to -1.08)	−2.1 (-3.3 to -0.82)	0.88
Fatigue	−3.1 (-4.2 to -2.01)^‡^	−4.0 (-5.5 to -2.6)	−2.0 (-3.6 to -0.32)	0.065	−4.3 (-5.6 to -2.9)	−1.6 (-3.2 to -0.12)	***0.012***
Sleep Disturbance	−2.2 (-3.4 to -0.94)^‡^	−2.6 (-4.3 to -0.95)	−1.6 (-3.5 to 0.24)	0.43	−2.9 (-4.4 to -1.3)	−1.3 (-3.0 to 0.42)	0.18
Ability to Participate in Social Roles and Activities^†^	2.9 (2.05 to 3.8)^‡^	3.6 (2.4 to 4.7)	2.1 (0.84 to 3.4)	0.11	3.8 (2.7 to 5.0)	1.8 (0.55 to 3.0)	***0.019***
Pain Interference^†^	−3.5 (-4.4 to -2.5)^‡^	−3.8 (-5.1 to -2.6)	−3.0 (-4.4 to -1.5)	0.36	−4.0 (-5.3 to -2.8)	−2.7 (-4.1 to -1.3)	0.16
Pain Intensity^†^	−1.0 (-1.3 to -0.73)^‡^	−0.94 (-1.3 to -0.57)	−1.08 (-1.5 to -0.66)	0.62	−0.95 (-1.3 to -0.59)	−1.06 (-1.5 to -0.65)	0.7

* All raw domain scores except Pain Intensity are standardized to a mean of 50 and an SD of 10.

† Data not available for all subjects.

‡ Paired *t* test *P* < 0.001.

§ Unadjusted-analysis *P* values correspond to ANOVA.

¶ Adjusted-analysis *P* values correspond to ANCOVA and adjust for pre-SMA PROMIS-57 domain subscore.

### Changes in Opioid Use

As part of their pain management, 44% of patients used prescribed opioid medication(s) at any time during the study period. We have determined the changes in opioid use in MMEs before and after participation in the SMAs. The average monthly MMEs decreased nonsignificantly from baseline (6 months before completion of the eighth/final SMA) to 6 months and 7–12 months after the completion of the eighth/final SMA (-49.8, -57.3, and -41.3, respectively) (Table 4). However, after participation in the SMAs ended, the use of opioid MMEs gradually went back to baseline. Patients who used more than 100 mg of average monthly dose at the first SMA (N = 18) decreased their use by 42% at the last (visit 8) SMA and below 50% of baseline for the remaining 12 months (data not shown). None of these positive changes, however, were statistically significant.

**Table 4.** Change in opioid dosage in MMEs before and after SMAs among opioid-use patients (N = 79)

Opioid Average Monthly Dosage Differences	Mean (95% CI)	*P* Value*
6 mo before first SMA to 6 mo after eighth/final SMA	−57.3 (-121.9 to 7.2)	0.08
6 mo before first SMA to 7–12 mo after eighth/final SMA	−41.3 (-112.2 to 29.6)	0.25
6 mo before first SMA to completion of eighth/final SMA	−49.8 (-115.09 to 15.4)	0.13
Completion of eighth/final SMA to 6 mo after eighth/final SMA	−7.5 (-38.4 to 23.4)	0.63
Completion of eighth/final SMA to 7–12 mo after eighth/final SMA	8.5 (-30.9 to 47.9)	0.67

* Paired *t* test.

## Discussion

Overall, this investigation showed that evidence-based, integrative, nonpharmacological therapies delivered via SMAs are an alternative, health-promoting approach to treating patients with chronic pain. Although the focus of this study was not to evaluate individual or specific therapeutic approaches (i.e., acupuncture, massage, yoga, or hypnotherapy/meditation), it may be argued that comprehensive use of such therapies contributed to self-reported improvements across multiple PROMIS domains without an increased use of opioid medications. Rather, we report a trend of decreased use of opioid medications by participants in these SMAs.

The findings in our study provide additional support for previous reports [21–25] that delivery of integrative medicine modalities via SMAs benefits patients with chronic pain in multiple ways. Although the composition, combinations, intensity, and delivery of individual integrative medicine therapies within SMAs have differed among the studies reported to date, they all beneficially complemented current management of patients with chronic pain. The structure of our SMAs, to our knowledge, included the largest spectrum of integrative and self-care therapies for patients with chronic pain, with acupuncture, yoga, self-massage, and hypnotherapy or meditation used at every SMA, plus an additional number of modalities incorporated throughout the SMAs (Figure 2 and Supplementary Data).

The most pronounced degree of beneficial pre–post changes for all evaluated patients was in the PROMIS Pain Interference (PI) and Fatigue domains (mean changes of -3.5 and -3.1, respectively) (Table 3). The mean pain intensity on the Likert scale decreased by 1.0 (from 6.2 to 5.2) (not shown; see also Table 2 for mean changes), similar to the value reported recently for patients with chronic pain and depression who were randomly assigned to participate in integrative medical group visits or primary care provider visits [22].

The PROMIS-PI domain is used to assess how pain might compromise daily activities and to evaluate the negative effects of pain on function in the range experienced by most patients with pain. The PROMIS-PI domain is highly correlated with coping strategies [26], similar to pain catastrophizing [27], and it may be superior to the Likert pain scale for pain assessment in some patients with foot, ankle, and spine pain [28, 29].

Although the pre–post changes reported by our patients indicate statistically significant improvements in all PROMIS-57 domains (see Table 3), uncertainty exists about what change in PROMIS scores represents a minimal clinically important difference in patients with pain [30, 31]. For example, for patients with chronic low back and musculoskeletal pain (who reported worse pain than the U.S. population norm; i.e., their mean PROMIS-PI scores were one SD above the population norm of 50 and thus similar to our patient population), the minimally important difference in the PROMIS-PI domain (including the eight-item scale used in our study) ranged from 2.0 to 3.0 points [32]. Their study results suggest that the mean improvement in the PROMIS-PI domain was not only statistically significant but also clinically significant, even when sensitivity analyses, including incomplete and dropout sets, were taken into account (Supplementary Data).

Similarly, the PROMIS Fatigue and Social domains for all patients improved, with a mean decrease of 3.1 and a mean increase of 2.9, respectively, while even larger changes were seen among patients who did not use opioid medications (-4.0 and 3.6, respectively). This did not change when adjusted for pre-SMA PROMIS-57 domain subscores (Table 3). Such a degree of beneficial change is thus likely to be clinically relevant. The minimally important difference for the Fatigue domain has been reported to range from 2.5 to 5.0 [31].

For patients with chronic musculoskeletal pain and comorbid depression and/or anxiety, the mean estimated minimally clinically important difference for the PROMIS eight-item Anxiety scale ranged from 1.7 to 5.0 [33], whereas our patients reported mean decreases in the Anxiety and Depression domains of 2.5 and 2.1, respectively. That would suggest that improvements in anxiety, especially for patients using no opioid medications (Table 3), are likely to be above the threshold that is meaningful to patients [31] even after multiple imputation analysis for all patients (Supplementary Data). Changes in depression, however, did not reach the minimal clinically important difference.

Improvements in multiple domains of PROMIS-57 in our patients occurred in spite of the trend toward decreased use of opioid medications (Table 4). The loss of long-term maintenance of decreased opioid use after the completion of the SMAs suggests that continued attendance at SMAs may be needed, although likely on a less frequent basis than once per week. Continued follow-up of our cohort and a larger number of patients would be needed to explore the possibility that a longer duration or higher intensity of intervention or additional follow-up via telehealth or e-coaching is required to reduce the use of opioids in a sustainable fashion.

Intensive inpatient (about 140 hours over 4 weeks) and outpatient (120 to 180 hours over 3 to 4 weeks) programs for patients with chronic non–cancer-related pain reported sustained decreases in opioid use at 6 and 12 months, whereas shorter outpatient programs (24 hours over 8 weeks) were less effective [34]. A recent systematic review of the literature suggests positive preliminary evidence that integrative medicine approaches can help reduce opioid use [35]. Although in our study, opioid use in MMEs decreased by 50 points during the SMAs as compared with the mean MME dose 6 months before the SMAs, such a decrease was statistically nonsignificant, and it was not sustained 6 or 12 months after the SMAs (Table 4).

Our study has several limitations. First, this is a pre–post study rather than a controlled study. Without a control group, we cannot know how the observed outcomes would compare with usual care. It is possible that potential patients in a control group would improve without any of the nonpharmacological interventions used in our studies as a result of simply being in the group setting and possibly as a result of the placebo effect [13, 36, 37]. It was recently reported in a controlled study [22] that patients in the control group reported multiple pre–post improvements, with no significant differences between the control (visits with the primary care physician) and the intervention groups (several integrative modalities), except in reducing emergency department visits and pain medication use.

We cannot know whether several of the observed statistically significant improvements are a direct result of patients’ participation in the SMAs, whether the degree of positive improvements is clinically meaningful, or whether those would translate into improvements in clinical outcomes that would be sustained in the long term. Considering data from the literature, we suggest that the PROMIS scores of our patients with chronic pain were worse than those of similar populations.

For example, when the baseline PROMIS-57 domains of patients with chronic pain in our study were compared with those reported in the literature, it seems that we had a population of patients with worse scores in the PROMIS-57 Physical Function (PF) and Pain Interference (PI) domains than postoperative PROMIS scores reported in the orthopedic literature [38] and the scores of patients in pain of spinal origin before and after surgery [39, 40]. For example, the mean PF score of our patients was 36.6 (that is, 13.4 points below the normative population), whereas in 13 of 14 orthopedic studies the PF score ranged from 42.6 to 66.4, with only one study with a score less than 40. Similarly, our patient population had a worse mean PROMIS-PI score (65.2—that is, 15.2 points above the normative population) than reported for postoperative orthopedic patients (ranging from 47.3 to 55.5, with only one study above 60) [38].

Second, our study focused only on the evaluation of patients who completed a PROMIS-57 questionnaire at the first and last group appointments; thus, we did not consider those who did not complete the questionnaires but who could have nevertheless benefited from their participation in the SMAs. Regardless, sensitivity analyses incorporating the excluded subjects were consistent across all scenarios, with the magnitude of change being smaller but remaining significant when last observation carried forward was used (Supplementary Data).

Third, we did not specifically correlate PROMIS-57 domain score changes with specific chronic pain diagnosis because our aim was not to evaluate outcomes for a specific pain condition. We also did not consider the influence of comorbid conditions our patients suffering from chronic pain may have had that could affect the interpretation of the PROMIS domains. A larger number of patients would be needed for a meaningful evaluation of that kind.

No PROMIS-57 evaluations were conducted at 6 and 12 months after the completion of the SMAs, and thus it is not clear whether the multiple benefits of the comprehensive intervention were sustained or whether they followed the trend observed for the opioid medication use. Opioid use was obtained from physician prescription orders rather than from filled prescriptions as documented in the electronic medical records and was not verified by blood or urine testing.

This study was conducted in a community-based ambulatory setting in the United States, and we cannot know whether the results are generalizable to other health care settings. The system, however, is similar to many health care delivery systems in the nation in terms of payer mix and provider reimbursement; thus, these outcomes would likely translate to other similar settings. Considering the limited demographic and clinical factors for our quite homogenous study population, we did not evaluate the associations of sociodemographic and health characteristics with clinical improvement within specific PROMIS subscores. A larger and more diverse population would be needed to obtain meaningful findings and avoid missing key predictors.

In conclusion, implementation of a comprehensive, multimodality, evidence-based, integrative and self-care nonpharmacological intervention is beneficial for patients with chronic pain in reducing pain and in improving multiple domains of their lives without increased use of opioid pain medications. Our study adds to a growing body of evidence that a combination of several complementary nonpharmacological therapies can be effectively delivered via SMAs. In the ongoing movement toward value-based health care, this type of intervention may enhance the transition away from the current, largely medication-based treatment options for patients with chronic pain.

## Authors’ Contributions

Statistical analysis and interpretation of data were conducted by AT, JD, SMD, MG, and RL. Critical revision of the manuscript for important intellectual content was performed by JZ, KNK, MFR, AT, DS, SMD, MG, and RL.

.

## Supplementary Data

Supplementary Data may be found online at http://painmedicine.oxfordjournals.org.

## Supplementary Material

pnaa418_Supplementary_Data

## Notes

Funding sources: This study was supported through internal institutional funds from the Center for Integrative and Lifestyle Medicine, Cleveland Clinic Community Care, and the Center for Population Health Research, Department of Quantitative Health Sciences, Cleveland Clinic..
