# Supplementary material for: “Living Well with Chronic Pain”: Integrative Pain Management via Shared Medical Appointments
Source: Pain Med. 2021 Feb 4;22(1):181–90. doi: 10.1093/pm/pnaa418 (PMC7861469; doi:10.1093/pm/pnaa418)
Supplement: pnaa418_Supplementary_Data [file pnaa418_supplementary_data.docx]

| **Supplemental Table 1. Comparison of analysis, incomplete data and drop-out sets** | | | | | | | |
| --- | --- | --- | --- | --- | --- | --- | --- |
|  | **Analysis set** | | **Incomplete data** | | **Drop-outs** | |  |
|  | **(N=178)** | | **(N=60)** | | **(N=44)** | |  |
| **Factor** | **N missing** | **Statistics** | **N missing** | **Statistics** | **N missing** | **Statistics** | **p-value** |
| Race, n (%) | 10 |  | 6 |  | 5 |  | 0.60^d^ |
| White |  | 120 (71.4) |  | 41 (75.9) |  | 24 (61.5) |  |
| Black or African American |  | 44 (26.2) |  | 12 (22.2) |  | 14 (35.9) |  |
| Other Race |  | 4 (2.4) |  | 1 (1.9) |  | 1 (2.6) |  |
| Age, median [Q1, Q3] | 4 | 62.0 [53.0, 69.0] | 0 | 58.0 [46.5, 68.0] | 8 | 53.5 [39.5, 64.5] | ***0.003^b^*** |
| Highest level of education completed, n (%) | 6 |  | 3 |  | 2 |  | 0.91^c^ |
| High school/GED or less |  | 18 (10.5) |  | 7 (12.3) |  | 4 (9.5) |  |
| Some college, no degree |  | 39 (22.7) |  | 14 (24.6) |  | 9 (21.4) |  |
| Associate or 2-year technical  degree / vocational school |  | 25 (14.5) |  | 11 (19.3) |  | 10 (23.8) |  |
| Bachelor's Degree |  | 53 (30.8) |  | 15 (26.3) |  | 10 (23.8) |  |
| Graduate Degree |  | 37 (21.5) |  | 10 (17.5) |  | 9 (21.4) |  |
| Current employment status, n (%) | 6 |  | 2 |  | 4 |  | ***0.004^c^*** |
| Employed |  | 47 (27.3) |  | 13 (22.4) |  | 14 (35.0) |  |
| Unemployed |  | 45 (26.2) |  | 30 (51.7) |  | 13 (32.5) |  |
| Retired |  | 80 (46.5) |  | 15 (25.9) |  | 13 (32.5) |  |
| Gender, n (%) | 5 |  | 0 |  | 11 |  | 0.47^c^ |
| Male |  | 23 (13.3) |  | 10 (16.7) |  | 7 (21.2) |  |
| Female |  | 150 (86.7) |  | 50 (83.3) |  | 26 (78.8) |  |
| Tobacco use, n (%) | 6 |  | 2 |  | 3 |  | ***0.007^d^*** |
| Past smoker |  | 68 (39.5) |  | 15 (25.9) |  | 6 (14.6) |  |
| Non-smoker |  | 99 (57.6) |  | 39 (67.2) |  | 34 (82.9) |  |
| Current smoker |  | 5 (2.9) |  | 4 (6.9) |  | 1 (2.4) |  |
| BMI, median[Q1, Q3] | 37 | 30.9 [26.1, 37.2] | 3 | 30.1 [23.8, 34.5] | -- | -- | 0.36^b^ |
| Opioid use, n (%) | 0 | 79 (44.4) | 0 | 32 (53.3) | 0 | 24 (54.5) | 0.31^c^ |
| *Pre-SMA PROMIS-57 subdomain scores* |  |  |  |  |  |  |  |
| Physical Function t-score, mean ± sd | 0 | 36.6 ± 5.9 | 2 | 37.4 ± 5.9 | 1 | 37.1 ± 6.0 | 0.65^a^ |
| Anxiety t-score, mean ± sd | 0 | 58.0 ± 9.7 | 2 | 59.3 ± 10.2 | 1 | 59.5 ± 8.7 | 0.48^a^ |
| Depression t-score, mean ± sd | 0 | 53.8 ± 8.4 | 3 | 55.2 ± 9.3 | 1 | 54.7 ± 8.7 | 0.56^a^ |
| Fatigue t-score, mean ± sd | 0 | 60.9 ± 9.2 | 2 | 62.9 ± 9.7 | 1 | 62.3 ± 9.7 | 0.30^a^ |
| Sleep Disturbance t-score, mean ± sd | 0 | 55.6 ± 8.4 | 3 | 59.3 ± 9.1 | 1 | 56.4 ± 8.3 | ***0.018^a^*** |
| Social t-score, mean ± sd | 0 | 41.0 ± 6.8 | 4 | 40.9 ± 8.8 | 1 | 40.6 ± 7.3 | 0.93^a^ |
| Pain Interference t-score, mean ± sd | 0 | 65.2 ± 5.9 | 3 | 65.9 ± 6.2 | 1 | 65.4 ± 6.1 | 0.73^a^ |
| Pain Intensity, mean ± sd | 0 | 6.2 ± 1.7 | 3 | 6.5 ± 2.0 | 1 | 6.0 ± 1.9 | 0.49^a^ |
| p-values: a=ANOVA, b=Kruskal-Wallis test, c=Pearson's chi-square test, d=Fisher's Exact test. | | | | | | | |

| **Supplemental Table 2a. Change of PROMIS-57 score before/after SMA among all patients, multiple imputation and last observation carried forward** | | | | |
| --- | --- | --- | --- | --- |
| **PROMIS-57 domain subscore (post – pre) **** | **Multiple Imputation** | | **Last Observation Carried Forward** | |
|  | **Mean (95% CI)** | **p-value*** | **Mean (95% CI)** | **p-value*** |
| **Analysis Set (N = 178)** | | | | |
| Physical Function | 1.3 (0.79, 1.9) | **<0.001** | 1.3 (0.79, 1.9) | **<0.001** |
| Anxiety | -2.5 (-3.5, -1.4) | **<0.001** | -2.5 (-3.5, -1.4) | **<0.001** |
| Depression | -2.1 (-3.01, -1.3) | **<0.001** | -2.1 (-3.01, -1.3) | **<0.001** |
| Fatigue | -3.1 (-4.2, -2.01) | **<0.001** | -3.1 (-4.2, -2.01) | **<0.001** |
| Sleep Disturbance | -2.1 (-3.4, -0.89) | **<0.001** | -2.2 (-3.4, -0.93) | **<0.001** |
| Ability to Participate in Social Roles and Activities | 3.0 (2.1, 3.8) | **<0.001** | 2.9 (2.03, 3.7) | **<0.001** |
| Pain Interference | -3.5 (-4.4, -2.5) | **<0.001** | -3.4 (-4.4, -2.5) | **<0.001** |
| Pain Intensity | -0.99 (-1.3, -0.71) | **<0.001** | -0.99 (-1.3, -0.72) | **<0.001** |
| **Analysis + Incomplete Sets (N = 238)** | | | | |
| Physical Function | 1.3 (0.78, 1.8) | **<0.001** | 1.01 (0.59, 1.4) | **<0.001** |
| Anxiety | -2.6 (-3.6, -1.6) | **<0.001** | -1.8 (-2.6, -1.00) | **<0.001** |
| Depression | -2.3 (-3.2, -1.3) | **<0.001** | -1.6 (-2.3, -0.92) | **<0.001** |
| Fatigue | -3.1 (-4.3, -1.8) | **<0.001** | -2.3 (-3.2, -1.5) | **<0.001** |
| Sleep Disturbance | -2.5 (-3.9, -1.08) | **0.001** | -1.6 (-2.6, -0.69) | **<0.001** |
| Ability to Participate in Social Roles and Activities | 2.9 (1.9, 3.9) | **<0.001** | 2.1 (1.5, 2.8) | **<0.001** |
| Pain Interference | -3.7 (-4.6, -2.8) | **<0.001** | -2.6 (-3.3, -1.8) | **<0.001** |
| Pain Intensity | -0.93 (-1.3, -0.59) | **<0.001** | -0.74 (-0.95, -0.53) | **<0.001** |
| **Analysis + Incomplete + Drop-Out Sets (N = 282)** | | | | |
| Physical Function | 1.3 (0.76, 1.8) | **<0.001** | 0.86 (0.49, 1.2) | **<0.001** |
| Anxiety | -2.5 (-3.6, -1.5) | **<0.001** | -1.5 (-2.2, -0.84) | **<0.001** |
| Depression | -2.1 (-3.1, -1.2) | **<0.001** | -1.3 (-1.9, -0.77) | **<0.001** |
| Fatigue | -3.0 (-4.1, -1.8) | **<0.001** | -2.0 (-2.7, -1.2) | **<0.001** |
| Sleep Disturbance | -2.5 (-4.3, -0.75) | **0.009** | -1.4 (-2.2, -0.58) | **<0.001** |
| Ability to Participate in Social Roles and Activities | 2.8 (1.9, 3.8) | **<0.001** | 1.8 (1.2, 2.4) | **<0.001** |
| Pain Interference | -3.6 (-4.5, -2.7) | **<0.001** | -2.2 (-2.8, -1.5) | **<0.001** |
| Pain Intensity | -0.88 (-1.3, -0.48) | **<0.001** | -0.62 (-0.80, -0.44) | **<0.001** |
| * paired t-test | | | | |
| ** All raw domain scores except Pain Intensity are standardized to with mean of 50 and a standard deviation of 10 | | | | |
| Multiple imputation results based on 5 imputation sets | | | | |
| Pre-SMA domain subscore carried forward for last observation carried forward results (assuming no change) | | | | |

| **Supplemental Table 2b. Change of PROMIS-57 score before/after SMA among patients without and with use of opioid medications, multiple imputation and last observation carried forward** | | | | | | |
| --- | --- | --- | --- | --- | --- | --- |
| **PROMIS-57 domain subscore (post – pre), Mean (95% CI) **** | **Multiple Imputation** | | | **Last Observation Carried Forward** | | |
|  | **No Opioids** | **Opioids** | **p-value** | **No Opioids** | **Opioids** | **p-value** |
| **Analysis Set (N = 178)** | | | | | | |
| Physical Function | 1.6 (0.86, 2.3) | 1.02 (0.19, 1.9) | 0.31 | 1.6 (0.86, 2.3) | 1.02 (0.19, 1.9) | 0.31 |
| Anxiety | -2.8 (-4.0, -1.6) | -2.0 (-3.4, -0.66) | 0.42 | -2.8 (-4.0, -1.6) | -2.0 (-3.4, -0.66) | 0.42 |
| Depression | -2.2 (-3.3, -1.08) | -2.1 (-3.3, -0.82) | 0.88 | -2.2 (-3.3, -1.08) | -2.1 (-3.3, -0.82) | 0.88 |
| Fatigue | -4.3 (-5.6, -2.9) | -1.6 (-3.2, -0.12) | ***0.012*** | -4.3 (-5.6, -2.9) | -1.6 (-3.2, -0.12) | ***0.012*** |
| Sleep Disturbance | -2.8 (-4.4, -1.3) | -1.3 (-3.0, 0.42) | 0.2 | -2.9 (-4.4, -1.3) | -1.3 (-3.0, 0.43) | 0.18 |
| Ability to Participate in Social Roles and Activities | 3.9 (2.8, 5.0) | 1.8 (0.54, 3.0) | ***0.013*** | 3.8 (2.7, 4.9) | 1.8 (0.55, 3.0) | ***0.022*** |
| Pain Interference | -4.1 (-5.4, -2.9) | -2.6 (-4.0, -1.2) | 0.11 | -4.0 (-5.2, -2.8) | -2.7 (-4.0, -1.3) | 0.15 |
| Pain Intensity | -0.95 (-1.3, -0.59) | -1.04 (-1.5, -0.63) | 0.75 | -0.95 (-1.3, -0.59) | -1.04 (-1.4, -0.63) | 0.75 |
| **Analysis + Incomplete Sets (N = 238)** | | | | | | |
| Physical Function | 1.6 (0.82, 2.3) | 0.99 (0.18, 1.8) | 0.32 | 1.3 (0.68, 1.8) | 0.74 (0.12, 1.4) | 0.23 |
| Anxiety | -3.0 (-4.4, -1.7) | -2.1 (-3.8, -0.33) | 0.45 | -2.2 (-3.2, -1.2) | -1.4 (-2.5, -0.33) | 0.31 |
| Depression | -2.4 (-3.6, -1.1) | -2.1 (-3.6, -0.68) | 0.8 | -1.7 (-2.6, -0.78) | -1.5 (-2.4, -0.56) | 0.8 |
| Fatigue | -4.3 (-5.7, -2.9) | -1.7 (-3.6, 0.33) | ***0.036*** | -3.4 (-4.5, -2.3) | -1.1 (-2.3, 0.05) | ***0.006*** |
| Sleep Disturbance | -3.2 (-4.9, -1.5) | -1.7 (-3.4, 0.02) | 0.18 | -2.2 (-3.4, -0.99) | -0.97 (-2.3, 0.34) | 0.17 |
| Ability to Participate in Social Roles and Activities | 3.9 (2.8, 5.0) | 1.7 (0.06, 3.4) | ***0.043*** | 2.9 (2.0, 3.8) | 1.3 (0.31, 2.2) | ***0.013*** |
| Pain Interference | -4.4 (-5.6, -3.2) | -2.9 (-4.5, -1.4) | 0.17 | -3.1 (-4.1, -2.2) | -1.9 (-2.9, -0.86) | 0.092 |
| Pain Intensity | -0.90 (-1.4, -0.42) | -0.98 (-1.5, -0.47) | 0.81 | -0.73 (-1.02, -0.44) | -0.75 (-1.05, -0.44) | 0.94 |
| **Analysis + Incomplete + Drop-Out Sets (N = 282)** | | | | | | |
| Physical Function | 1.6 (0.93, 2.3) | 0.93 (0.16, 1.7) | 0.19 | 1.09 (0.59, 1.6) | 0.60 (0.08, 1.1) | 0.18 |
| Anxiety | -2.9 (-4.3, -1.6) | -2.1 (-4.1, -0.02) | 0.51 | -1.9 (-2.8, -1.06) | -1.08 (-2.0, -0.16) | 0.18 |
| Depression | -2.2 (-3.6, -0.90) | -2.0 (-3.6, -0.53) | 0.85 | -1.5 (-2.3, -0.71) | -1.2 (-2.0, -0.39) | 0.59 |
| Fatigue | -4.3 (-5.7, -2.9) | -1.5 (-3.4, 0.37) | ***0.028*** | -3.0 (-3.9, -2.0) | -0.86 (-1.8, 0.13) | ***0.003*** |
| Sleep Disturbance | -3.3 (-5.2, -1.4) | -1.7 (-3.8, 0.49) | 0.14 | -2.0 (-3.0, -0.93) | -0.72 (-1.8, 0.39) | 0.1 |
| Ability to Participate in Social Roles and Activities | 4.0 (3.0, 5.0) | 1.6 (-0.07, 3.2) | ***0.015*** | 2.5 (1.8, 3.3) | 1.01 (0.21, 1.8) | ***0.008*** |
| Pain Interference | -4.5 (-5.6, -3.4) | -2.7 (-4.2, -1.2) | 0.075 | -2.7 (-3.6, -1.9) | -1.5 (-2.4, -0.66) | 0.061 |
| Pain Intensity | -0.87 (-1.4, -0.36) | -0.90 (-1.5, -0.24) | 0.95 | -0.65 (-0.89, -0.40) | -0.60 (-0.86, -0.34) | 0.79 |
| ** All raw domain scores except Pain Intensity are standardized to with mean of 50 and a standard deviation of 10 | | | | | | |
| All results adjust for pre-SMA PROMIS-57 domain subscore and p-values correspond to ANCOVA | | | | | | |
| Multiple imputation results based on 5 imputation sets | | | | | | |
| Pre-SMA domain subscore carried forward for last observation carried forward results (assuming no change) | | | | | | |

**Supplemental Material 3. Cleveland Clinic Pain Shared Medical Appointment (SMA): Manual of Sessions**

**Week 1:**

***Vitals and brief physical exam:*** This is done as patients enter the SMA room. They will also have an intro to the program in week 1 and sign an acupuncture release. Patients are encouraged to try everything during the next 8 weeks and to focus on creating a personal plan to address their discomfort.

***Check-in process:*** Done as a group, with the holistic psychotherapist and physician facilitating while patients answer a

specific set of questions based on their experiences and their use of materials presented in the program. This is where the physician is able to document the subjective part of their visit. The primary goal here is to assess the efficacy of their

experiences with the various modalities.

***Lecture:*** Acupressure for pain reduction. The acupuncturist for the program gives a lecture on acupressure and teaches the participants how to identify areas specific to their location of pain. Participants have an opportunity to practice using the acupressure techniques and ask questions about their current need.

***Acupuncture:*** Each participant receives the same acupuncture, based on NADA protocol, at each session. They are in a group acupuncture room.

***Hypnotherapy and Guided Meditation:*** This is led by the therapist while the patients are in the acupuncture room. Each

week a different hypnotherapy script is utilized. The goal for the hypnotherapy is to assist the patient in creating new

patterns of thought that are in alignment with their goals of decreasing physical and emotional discomfort in their lives. The script allows space to add in particular positive suggestions based on what was shared during the daily check in phase.

**Chair yoga and gentle stretching**: At the end of each shared medical appointments

**Week 2:**

***Vitals and brief physical exam:*** This is done as patients enter the SMA room and it is the same at each appointment

***Check-in process:*** Done as a group, with the holistic psychotherapist and the physician facilitating while patients answer a specific set of questions based on their experiences and their use of materials presented in the program. This is where the physician is able to document the subjective part of their visit and assess what modalities are working for the patient.

***Lecture:*** Massage for chronic pain. A brief lecture is given by a massage therapist on the clinical importance of massage and then each participant receives a short chair massage within the SMA room.

***Acupuncture:*** as above, the same at each appointment.

***Hypnotherapy and Guided Meditation:*** This is led by the holistic psychotherapist while the patients are in the acupuncture room. Each week is a different topic, with week 2 related to creating a positive mind shift.

***Chair yoga and gentle stretching***: The same as in the previous group appointment.

**Week 3:**

***Vitals and brief physical exam:*** The same as in the previous group appointment.

***Check-in process:*** Done as a group, with the holistic psychotherapist and the physician facilitating while patients answer a specific set of questions based on their experiences and their use of materials presented in the program. This is where the physician is able to document the subjective part of their visit and assess what modalities are working for the patient. The therapist is also listening to the language of each patient and assisting them with cognitive restructuring.

***Lecture:*** Reducing pain through nutrition. A nutritionist led medical discussion about an anti-inflammatory diet and how

those foods can help to reduce pain in the body.

***Acupuncture:*** The same as in the previous group appointment.

***Hypnotherapy and Guided Meditation:*** This is led by the therapist while the patients are in the acupuncture room. Each

week is a different topic related to patient content from Check in.

***Chair yoga and gentle stretching***: The same as in the previous group appointment.

**Week 4:**

***Vitals and brief physical exam:*** The same as in the previous group appointment.

***Check-in process:*** Done as a group, with the holistic psychotherapist and the physician facilitating while patients answer a specific set of questions based on their experiences and their use of materials presented in the program. This is where the physician is able to document the subjective part of their visit and assess what modalities are working for the patient. Patients are asked to identify at least 2 modalities that are helpful to the reduction of their discomfort.

***Lecture:*** Chiropractic Care for pain control. A chiropractor leads this medical discussion and helps the patients to understand how everyday positions can contribute to the pain issues they already have and then teaches them how to modify those positions to improve discomfort.

***Acupuncture:*** The same as in the previous group appointment.

***Hypnotherapy and Guided Meditation:*** This is led by the therapist while the patients are in the acupuncture room. Each

week is a different topic, with week 4 related to patient needs. Breathing, relaxation and visualization are utilized to support the patient's cognitive restructuring.

***Chair yoga and gentle stretching***: The same as in the previous group appointment.

**Week 5:**

***Vitals and brief physical exam***: The same as in the previous group appointment.

***Check-in process:*** Done as a group, with the holistic psychotherapist and the physician facilitating while patients answer a specific set of questions based on their experiences and their use of materials presented in the program. This is where the physician is able to document the subjective part of their visit and assess what modalities are working for the patient. Questions around sleeping patterns are explored in this week’s check in.

***Lecture:*** Understanding the mechanisms of pain. This is a physician led medical discussion about how inflammation, which comes from many different factors, increases pain and discomfort in the body.

***Acupuncture:*** The same as in the previous group appointment.

***Hypnotherapy and Guided Meditation:*** This is led by the therapist while the patients are in the acupuncture room. Each

week is a different topic, with week 5 related to the process of changing their mindset in order to change their life in a

positive manner.

***Chair yoga and gentle stretching***: The same as in the previous group appointment.

**Week 6:**

***Vitals and brief physical exam:*** The same as in the previous group appointment.

**Check-in process:** Done as a group, with the holistic psychotherapist and the physician facilitating while patients answer a specific set of questions based on their experiences and their use of materials presented in the program. This is where the physician is able to document the subjective part of their visit and assess what modalities are working for the patient.

***Lecture:*** The Holistic psychotherapist utilizes expressive arts therapy techniques to assist patients in exploring the mind body connection. The focus of this session is learning to express emotions in a healthy manner. Art, drumming and breath are used in this session.

***Acupuncture:*** The same as in the previous group appointment.

***Hypnotherapy and Guided Meditation***: This is led by the therapist while the patients are in the acupuncture room. Each

week is a different topic, with week 6 related to challenging old beliefs and patterns that are no longer serving them.

***Chair yoga and gentle stretching***: The same as in the previous group appointment.

**Week 7:**

***Vitals and brief physical exam:*** The same as in the previous group appointment.

***Check-in process:*** Done as a group, with the holistic psychotherapist and the physician facilitating while patients answer a specific set of questions based on their experiences and their use of materials presented in the program. This is where the physician is able to document the subjective part of their visit and assess what modalities are working for the patient.

***Lecture:*** Goal setting and building a healthy support community. Patients are introduced to techniques that support

themselves in reviving, sharing and receiving community support in the area of goal setting and positive focus. Participants learn to create positive partnerships for accountability and success.

***Acupuncture:*** The same as in the previous group appointment.

***Hypnotherapy and Guided Meditation:*** This is led by the therapist while the patients are in the acupuncture room. Each

week is a different topic, with week 7 focusing on healthy emotional expression and creating goals that motivate healthy

behavior change.

***Chair yoga and gentle stretching***: The same as in the previous group appointment.

**Week 8:**

***Vitals and brief physical exam:*** The same as previous group appointment.

**Check-in process:** Done as a group, with therapist and physician facilitating while patients answer a specific set of questions based on their experiences and their use of materials presented in the program. This is where the physician is able to document the subjective part of their visit and assess what modalities are working for the patient.

***Lecture:*** Chinese Herbal Therapy and Topical Treatments for pain control. This is a topic led by our Chinese herbal medicine practitioner. There is a medical discussion on how the Chinese herbal medicine clinic works, why it could be a helpful resource and how topical options for pain control are good, low risk options for managing pain sensations.

***Acupuncture:*** The same as previous group appointment.

***Hypnotherapy and Guided Meditation***: This is led by the therapist while the patients are in the acupuncture room. Each

week is a different topic, with week 8 related to utilizing the modalities in the program to support their healing and to

increase the quality of their life.

***Chair yoga and gentle stretching***: The same as in the previous group appointment.

Follow-up appointments: Every 4 weeks, former participants are invited back to the program to share their challenges and triumphs with the current participants. This encourages continued engagement and creates additional support networks outside of the group.
